# Reshaping de novo protein switches into bioresponsive materials for biomarker, toxin, and viral detection

**DOI:** 10.1002/adma.202208556

**Published:** 2023-01-13

**Authors:** Luciana d’Amone, Giusy Matzeu, Alfredo Quijano-Rubio, Gregory P. Callahan, Bradley Napier, David Baker, Fiorenzo G. Omenetto

**Affiliations:** 1 Silklab, Department of Biomedical Engineering, Tufts University; Medford, MA, USA.; 2 Laboratory for Living Devices, Tufts University; Medford, MA, USA.; 3 Department of Biochemistry, Institute for Protein Design, University of Washington; Seattle, WA, USA.; 4 Department of Bioengineering, University of Washington; Seattle, WA, USA.; 5 Howard Hughes Medical Institute, University of Washington; Seattle, WA, USA.; 6 Department of Electrical and Computer Engineering, Tufts University; Medford, MA, USA.; 7 Department of Physics, Tufts University; Medford, MA, USA.

## Abstract

*De novo* designed protein switches are powerful tools for specifically and sensitively detecting diverse targets with simple chemiluminescent readouts.^1,2^ Finding an appropriate material host to entrain these constructs without altering their thermodynamics would enable a variety of sensing formats to monitor exposure to pathogens, toxins, or for disease diagnosis. Here we generate a *de novo* protein-biopolymer hybrid that maintains the detection capabilities induced by the conformational change of the incorporated proteins in response to analytes of interest in multiple, shelf-stable material formats. A set of functional demonstrator devices including personal protective equipment such as masks and laboratory gloves, free-standing films, air quality monitors, and wearable devices is presented to illustrate the versatility of the approach. Such formats are designed to be responsive to human epidermal growth factor receptor (HER2), anti-hepatitis B (HBV) antibodies, Botulinum neurotoxin B (BoNT/B), and severe acute respiratory syndrome coronavirus 2 (SARS-CoV-2). This rationally designed combination of form and function offers wide opportunities for ubiquitous sensing in multiple environments by enabling a large class of bio-responsive interfaces of broad utility.

The development of shelf-stable, portable, modular sensing devices to identify biomarkers at low concentration levels often relies on transduction mechanisms of different degree of complexity, to achieve the necessary specificity, sensitivity, while limiting interference from other analytes.^3,4^ Refined methods based on biochemical transduction agents such as RNA or antibodies ^5^ have proven effective, yet the processing, form factors, and shelf-stability of these formats provide challenges in sample preparation and readout.

*De novo* designed protein-based biosensors have been recently demonstrated to be a versatile, reconfigurable biochemical transduction mechanism for sensing applications ^2,6,7^. In these systems, the sensing function is provided by the synergy of two designed protein components, the lucCage and the lucKey. The lucCage undergoes conformational changes (from a closed state to an open state) after interacting with a target molecule and includes a split luciferase fragment and an analyte-binding domain. The lucKey includes a key peptide that binds to the open state of the lucCage domain and the complementary split luciferase fragment. The association of the two protein components results in reconstitution of luciferase activity. The ensuing luciferase bioluminescence provides a rapid, specific, and sensitive readout of the analyte-driven lucCage–lucKey switch association^2,7^. The incorporation of *de novo* protein sensors into point-of-care devices would open promising avenues for the design of selective and versatile sensing units for routine monitoring of specific biomarkers.

Regenerated silk fibroin (RSF), derived from the native *Bombyx mori* silkworm fibers, has been intensively investigated because of its outstanding combination of biocompatibility, biodegradability, and mechanical flexibility.^8–10^ Various fabrication technologies have been used to transform a water-based suspension of silk fibroin into a variety of end material formats, including hydrogel, porous sponges, microneedles, and responsive biointerfaces^11–15^. Furthermore, RSF has been utilized to stabilize some intrinsically labile biomolecules, such as drugs^16–19^, enzymes^20–22^, pH indicators^23^, and complex biofluids^24^, enabling the fabrication of bioresponsive material formats with long shelf-life.

An evaluation of the functionality of the entrained *de novo* protein system in the various end formats of silk protein is necessary given the dependence of the Key-Cage sensitive and selective detection mechanism on the precisely tuned binding energies necessary to achieve activation at relevant target concentrations. This activation process, typically carried out in solution after refrigerated storage of the Key-Cage system, leads to a conformational transition that results in a detectable luminescence in response to analyte binding. The inclusion of carefully designed *de novo* protein systems in a self-assembling silk proteinaceous matrix undergoing phase and conformational transitions could perturb their energetic states and inhibit their sensing capability.

With the goal of generating a broad class of bio-responsive sensing material formats of broad utility to move beyond traditional colorimetric or electrochemical point of care devices ^25–28^, a number of bio-responsive demonstrator materials were assembled and analyzed by incorporating several Key-Cage protein systems directly into the liquid RSF. These RSF formulations were then used to generate a variety of material formats using inkjet printing, lyophilization, electrospinning, and film casting. The integration of dynamic, responsive protein switches with silk protein through various fabrication strategies provides smart substrates designed to respond to (i) the receptor-binding domain (RBD) in Severe Acute Respiratory Syndrome Coronavirus 2 (SARS-CoV-2) spike protein, (ii) the Human Epidermal Growth Factor Receptor 2 (HER2), (iii) the Botulinum Neurotoxin B (BoNT/B), and (iv) the Anti-Hepatitis B Virus antibody (anti-HBV) (Fig. 1).

## Key-Cage/RSF hybrids characterization and stability

Free-standing bioresponsive films and sponges were fabricated by adjusting the ratio of lucKey and lucCage within the RSF solution to tune material assembly, performance, and permeability (see Supporting Information for additional details). The viability of the *de novo* protein system contained in the solid material formats is assessed by dissolving both sponges and films in water and then exposing them to various analyte concentrations to evaluate the protein performance through its chemiluminescent response (Fig. 2a). For both formats, chemiluminescence is still detected with a signal intensity that is proportional to increasing target concentrations for all Key-Cage compounds used, underscoring the versatility and maintained reactivity of the *de novo* protein-biopolymer composite. Evaluation of the luminescent signal shows the ability to detect within minutes (~10-20 minutes) HER2, BoNT/B, and anti-HBV down to nM levels (Fig. 2b, 2d, 2h, and S2–S9) (Limit of Detection (LOD), LOD_HER2sponges_ = 1 nM, LOD_HER2films_ = 1.2 nM; LOD_BoNT/Bsponges_ = 2.4 nM, LOD _BoNT/Bfilm_ = 2.1 nM; LOD_anti-HBVsponges_ = 1.5 nM, LOD_anti-HBVfilms_ = 1.8 nM), and RBD down to pM levels (Fig. 2f)(LOD_sponges_ = 2 pM, LOD_films_ = 11 pM).

To evaluate the shelf stability of these materials, accelerated aging tests were performed on a subset of the bioresponsive material formats. RBD sponges and films were stored at 60°C for 4 months and their responsiveness were analyzed after specific intervals. Over the 4-month time frame, the sensing performance of these formats (both sensitivity and dynamic range) were preserved, with an LOD_avg_ of 16±7 pM for RBD sponges, and LOD_avg_ of 17±7 pM for RBD films (Fig. S13). HER2 sponges were also tested for stability after storage at room temperature (20-25ºC) for one year and resulted still responsive with sensitivity within the nM range (Fig. S14) (LOD = 5nM).

Bioresponsive films were also evaluated with an *in-situ* approach where films were directly exposed to the analyte and activated with furimazine to generate a chemiluminescent signal. This approach would provide advantages for use outside the laboratory and does not require complete dissolution of the sensing substrate, which would facilitate usage by untrained users. HER2 sensing films were exposed to various analyte concentrations and furimazine (Fig. 2c). The resulting luminescent image, analyzed to correlate intensity variations with changes in HER2 exposure, shows a linear response for concentration values between 20-500 nM (LOD = 15nM). Similarly, bioresponsive films were functionalized with lucCage-lucKey switches for BoNT/B (Fig. 2e), and RBD (Fig.2g, S11) and anti-HBV(Fig. 2i), demonstrating the possibility to generate free-standing sensing elements for a variety of targets.

Biosensors are prone to interference given the large amounts of non-specific interactions with background molecules, which can affect their sensing capabilities in biofluids. To further evaluate the RSF/Key-Cage hybrid response to different specimens, simulated saliva spiked with different concentrations of RBD was used to test the system’s performances in complex media. RBD was detected in simulated saliva down to 10 pM concentration without compromising the dynamic sensing range (Fig. 2g). Additionally, the specificity of RBD bioresponsive films was evaluated by testing their responsiveness to potentially interfering analytes (Fig. 2l). After exposure to furimazine and various targets in the nM range, the RBD films were active and responsive only in presence of the corresponding target (i.e., RBD protein) demonstrating the specificity of the RSF/Key-Cage hybrids.

## Sensing interfaces and wearable devices based on de novo proteins

RSF is advantageous because of its polymorphism that allows for processing and reshaping into different material composites that preserve the biological function of their constituents. As a proof of concept, a de novo protein/RSF solution hybrid was formulated to enable inkjet printing. The printed patterns serve as sensing elements and provide a chemiluminescent response that is directly proportional to the analyte concentration (Fig. 3). Shear forces and volume ejection that occur during printing did not alter the functional capacity of the printed format enabling a scalable manufacturing process for the development of ad hoc sensing surfaces. Demonstrator devices that leverage printing and recapitulate the specificity and sensitivity of the *de novo* systems are illustrated in Fig. 3 as breast cancer biomarkers detectors (Fig.3b–c), toxin (Botulin) detectors for food contamination (Fig.3d–e), and viral detectors (COVID, Fig. 3f–g). In all cases, the printed patterns are interfaced to various substrates, exposed to the analyte of interest, activated with furimazine, and evaluated for chemiluminescence intensity as a function of detected analyte concentration (Fig. 3a, additional details in S.I.).

For viral detection, RBD/RSF hybrid inks were printed on laboratory gloves and surgical masks. Exposure to RBD targets shows chemiluminescent response proportional to analyte concentration while maintaining the geometry of the printed pattern. The surgical mask provides a convenient complementary approach to nasopharyngeal swabs for the detection of SARS-COV-2 and were evaluated by spraying furimazine and RBD target to mimic breathing of potential infected users (Fig. 3a, f–g, S12A,B). Detected responses show a sensitivity down to concentrations of 0.25 nM on gloves and 5nM on the surgical mask. In the latter case, the absorbing behavior of the mask non-woven fabric affected the sensitivity of the functional ink. However, the sensing capability of de novo protein/RSF solution hybrids can be improved by fabricating non-woven meshes directly with the proteins hybrid solution via electrospinning for detection and small-pore size filtration (Fig. S11B). In this case, despite the harsh processing conditions (fiber shear forces, high voltages), the sensing performance was preserved overall within the 2-1250 pM concentration range.

A demonstrator format for detection of HER2 was formulated with a hybrid RSF/HER2 ink interfaced to a silicone breast pad. This sensing substrate was selected given the importance of HER-2 as a breast cancer biomarker in nipple fluids and can serve as a potential interface for early detection of breast cancer, efficacy of treatment, or to discriminate between benign or malignant discharge^29–31^. HER2 concentration is tracked directly on the dotted pattern dispensed on the silicone pad in a short timeframe (~ 20 minutes) and within the nM range (Fig. 3c, S12C).

Toxins relevant to food safety and human health such as botulin were also explored by using relevant de novo protein systems and hybridizing them with RSF to print patterns on the inside of a food jar lid. Similarly, to the previous example, the approach could allow for the detection of contamination, spoilage, or tampering of the contents by examination of the lid’s luminescence response in 20 minutes and at the toxin concentration within the nM range (Fig. 3e, S12D).

Printing provides a modular approach for the use of multiple Key-Cage systems across a variety of surfaces, adding utility to wearable interfaces, surfaces and containers, or other high-touch surfaces providing early warning against exposure to hazardous agents.

Additional material formats can enable alternative biosampling approaches that recapitulate the functional attributes of biopolymer matrices and de novo systems. High surface-to-volume porous formats can also be synthesized to sample air through bioresponsive bulk materials. A demonstration is shown by generating a viral-sensing drone, obtained by freeze-drying RBD/RSF composites in the shape of the top half of the ~3 cm aircraft (Fig.3h). The drone was exposed to contaminated aerosols in a small chamber (see SI for details). Analysis of the permeable, structural, bioresponsive material after exposure showed a response to the target of interest, opening for standoff environmental sampling and detection of viruses and toxins (Fig. 3i).

The hybrid combination of silk fibroin and *de novo* designed protein switches offers a variety of material formats that can be easily adapted to generate a wide number of sensing interfaces that can in turn be distributed and used in a multitude of environments that range from personal monitoring to point-of-care, to environmental sensing. For example, sensing sponge formats may be adopted within intensive care units to quantify the amount of air borne viral particles circulating in the room. Film-like devices sensitive to toxins variations may be of valuable use for water quality assessment in remote locations. Bio-reactive garments may enable the non-invasive monitoring of biological fluids (e.g., sweat, saliva) and unravel the presence of biomarkers of interest (e.g., cortisol, amylase) at lower concentrations than what is currently achievable with standard miniaturized sensing devices while offering broad utility in a practical and universally deployable material format.

## Methods

### 1. Silk fibroin isolation

Regenerated silk fibroin (RSF) solution was isolated from B. mori cocoons as previously reported ^1^.Briefly, silk cocoons were shredded, separated from impurities, and boiled in a 0.02 M solution of sodium carbonate to remove sericin. Boiling time was set to 120 minutes to fabricate bio-reactive films, sponges, and inks, and to 30 minutes to prepare mixes for electrospinning. The fibers were rinsed with deionized water three times for 20 minutes and air-dried overnight. The dried silk mats were subsequently dissolved in a 9.3 M solution of lithium bromide at 60°C for 4 hours. The chaotropic salt was then removed through dialysis (dialysis membranes MWCO 35 kDa) against deionized water for 48 hours, changing the water 6 times at regular intervals, which yielded a 7-8 wt % silk solution. The final solution was centrifuged at ∼12,700 × g for 20 minutes, filtered to separate remaining impurities, and stored at 4°C.

### 2. Preparation of silk-based sponges and films

LucCage and lucKey of desired concentrations were prepared from the initial stock solutions listed in Table S1. Aliquots of the lucCage and lucKey stock solutions were combined with silk solutions at 2 wt% or 4 wt%, to obtain sensing films and sponges with different concentrations, as reported in Table S2. To prepare bio-reactive film formats, 100 μL of the sensing mix was cast on PDMS substrates. Films were dried at room temperature (25°C) overnight. To prepare bio-reactive sponges, 100 μL of silk-based solution was aliquoted in PCR tubes, flash-frozen, and lyophilized overnight with a Labconco FreeZone 2.5 Liter Benchtop Freeze Dry System (USA). Bio-reactive films and sponges were stored in Eppendorf tubes at room temperature before undergoing further testing.

### 3. Preparation of silk-based inks

The lucKey-lucCageRBD stock solutions were diluted to the final concentration of 25 nM in 2 wt% silk fibroin solution using deionized water prefiltered with a 0.22 μm syringe filter. The solution was mixed with a plate shaker for 45 minutes to enable pre-equilibration and subsequently 0.2 wt% Tween 20 was added to yield the printing mix. 20 μL of silk ink was printed onto white polystyrene substrates, surgical masks, and nitrile laboratory gloves using an HP D300e Digital Dispenser and T8+ dispense head cassettes. The printing process was influenced by the viscosity of the solution, the surface tension, and the alignment between substrates and dispense head, which affected the dispensing accuracy. Furthermore, the hydrophobicity of the substrates impacted the resolution of the printed patterns. Although different types of surfaces were used, the thickness of the chosen substrates (1.6 mm) enabled high dispense accuracy and spot alignment during the printing process. The printed patterns were designed with the HP Bio Pattern software. The printing resolution was optimized by tuning the number of layers dispensed and the volumes dispensed per-spot. The final volume of the pattern was given by the total volume dispensed per-spot multiplied by the number of spots in the pattern. 25nM lucKey-lucCageHER2 and 50nM BoNT/B solutions were prepared and manually dispensed on a silicone breast pad in 2ul drops (HER2) or a jar lid in 20ul (BoNT/B).

### 4. Bioluminescence kinetics of films and sponges

Chemiluminescent readings were recorded with a Synergy HT Microplate Reader (BioTek, USA). The sensing silk-based sponges and films were dissolved in deionized water and mixed for 45 minutes before analysis. 15 μL of furimazine substrate (Nano-Glo luciferase assay reagent, Promega) was added to the sensing mix to obtain a final total volume of 2015 μL. 95 μL aliquots of the lucKey-lucCage silk solutions were transferred into a white polystyrene 96 well plate and the solution was monitored to obtain the baseline reading by acquiring the bioluminescence signal every 3 minutes for 50 minutes. After this time, 5 μL of serially diluted target proteins were added to the mix into the 96 well plate. The final solution was mixed for 5 minutes. Bioluminescence kinetics were recorded for 3 additional hours to acquire the response of the dissolved bio-reactive silk formats in presence of various analytes concentrations. The limit of detection (LOD) was calculated as the value related to the cross point between the extrapolation of the lines defining the nonresponsive range and linear-response range of the system.

### 5. Bioluminescence imaging

Bioluminescence images were acquired with the Molecular Imaginer^®^ ChemiDoc XRS + Imaging System (Bio-Rad) using the Chemi High Resolution mode. Images were acquired at time intervals of 5 minutes, for a total of 25 minutes.

### 6. Accelerated aging tests

Accelerated aging tests for the RBD bio-reactive films and sponges were performed to evaluate the intrinsic stability of the 2 wt% and 4 wt% silk formats embedding lucKey and lucCage at 1 and 5 nM over time (Figure S13). Samples were stored in an incubator at 60°C for a total of 4 months and analyzed after 7 hours, 2 days, 7 days, 2 months, and 4 months. More precisely, films and sponges were removed from the incubator and equilibrated at room temperature. They were then dissolved in deionized water and analyzed with the Synergy HT Microplate Reader following the same protocol described in Methods Section 4.

## Supplementary Material

De Novo Sensors_FIN_SI

## Figures and Tables

**Fig. 1:**
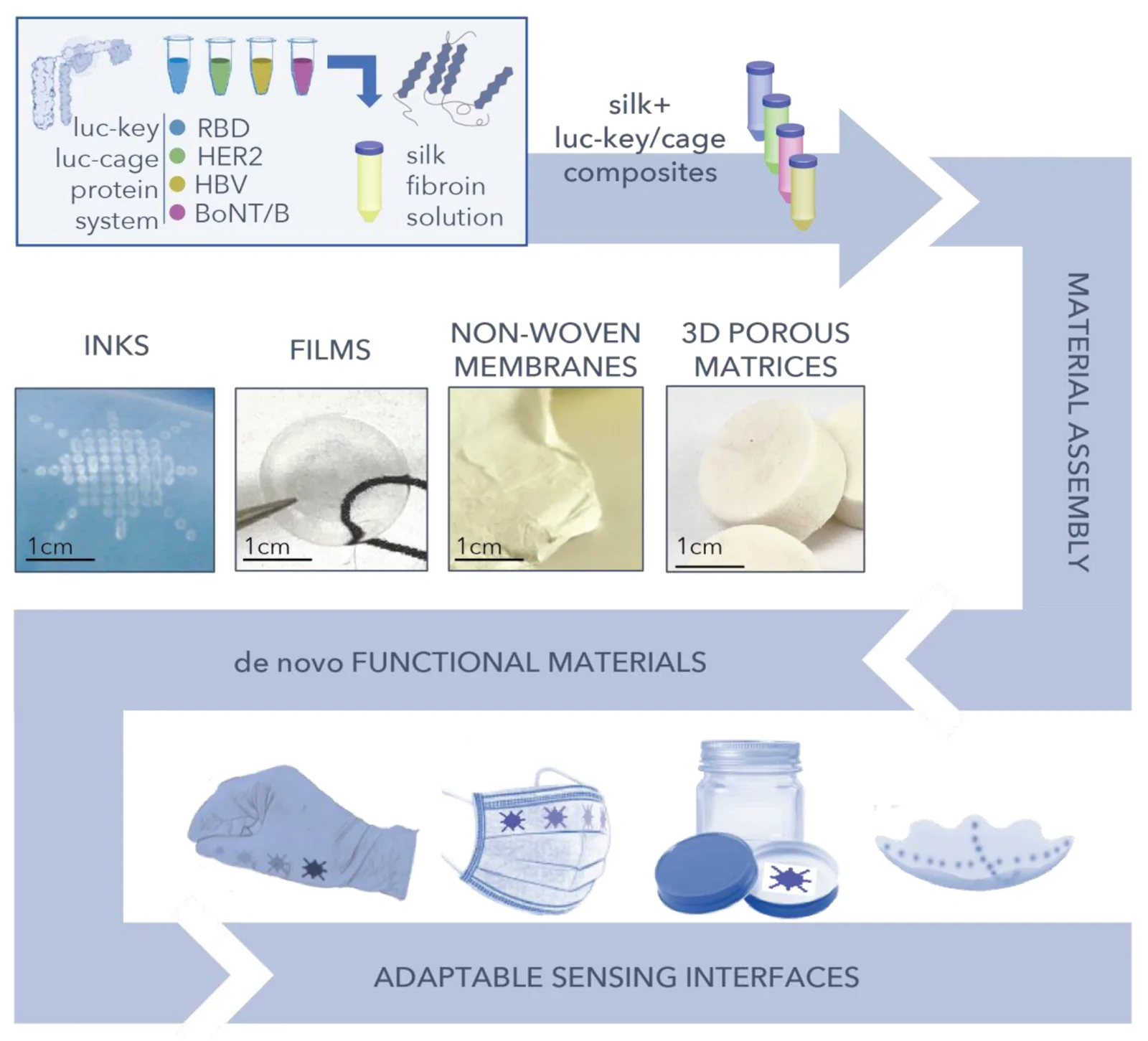
Fabrication of RSF biosensing formats for universal biomarker detection. Protein switches (lucKey-lucCage) that enable the detection of SARS-COV-2 spike protein (RBD), HER2 receptor (HER2), anti-hepatitis B antibodies (anti-HBV), and Botulinum Neurotoxin B (BoNT/B), are combined with RSF to fabricate a library of bio-active platforms, comprising free standing sponges and films, electrospun non-woven mats, bio-active personal protective equipment (mask and glove), jar lids, and breast pads capable of responding to a wide range of targets.

**Fig. 2:**
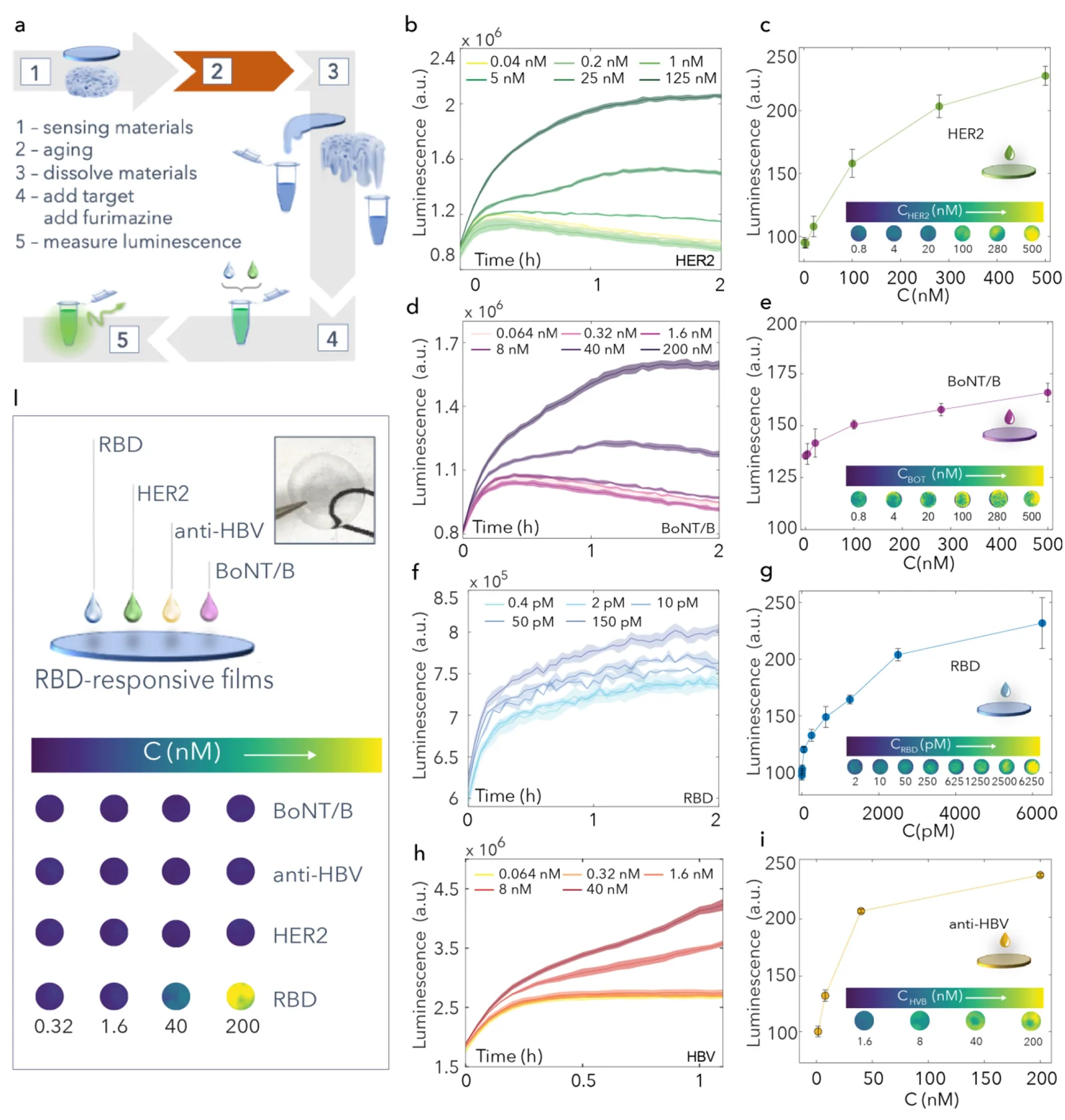
Characterization of silk sponges and films for biomarkers detection. **a**, Schematic representation of the characterization and stability studies procedures of de novo protein/RSF hybrid films and sponges. **b**, **d**, **f**, **h** Luminescence response of sponges prepared with 2% RSF and 5nM lucKey-lucCageHER2 (**b**), 25nM lucKey-lucKeyBoNT/B (**d**), 5nM lucKey-lucCageRBD (**f**), or 6.25nM lucKey-lucCageHBV (**h**) after dissolution and exposure at various concentrations of analytes. **c**, **e**, **g**, **i** Bioluminescent response (reported as gray value) of films prepared with 2% RSF 5nM lucKey-lucCageHER2 (**c**), 2% RFS 25 nM lucKey-lucKeyBoNT/B (**e**), 4% RSF 5nM lucKey-lucCageRBD (**g**), and 2% RSF 6.25 nM lucKey-lucCageHBV (**i**) as a function of target concentration. Insets represent the corresponding bioluminescence images in false color. (**l**) Analyte specificity of lucKey-lucCageRBD film. Schematic representation of testing procedure (top), photograph of RSF film (inset), and bioluminescence images (bottom) of 2% RSF 5nM lucKey-lucCageRBD recorded in presence of RBD target and potential interfering analytes (HER2, anti-HBV, or BoNT/B).

**Fig. 3:**
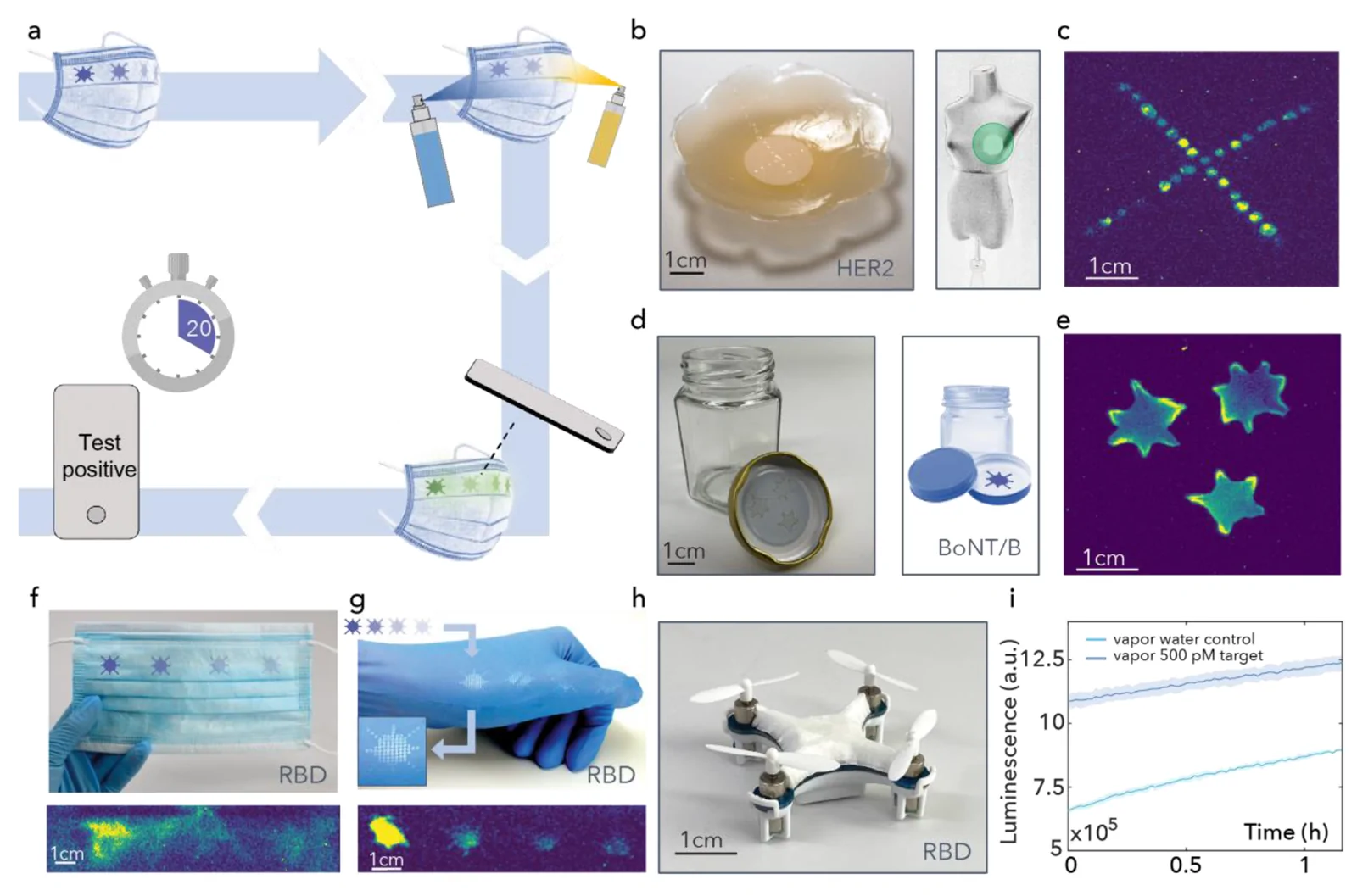
Sensing interfaces for the detection of different biomarkes. **a** Schematic representation of processing steps for RBD detection on a bioactive mask. **b**, Functionalized breast pads (left) can be used for HER2 detection in nipple fluids (right). **c**, Bioluminescence images of the functionalized breast pad in false color after exposure at various HER2 target concentrations (50 nM, 250 nM, 500 nM, and 12.5 nM clockwise from the top left). **d**, Functionalized lids can detect jars contaminated with BoNT/B. **e**, Bioluminescence images of the functionalized jar lid in false color after exposure at different BoNT/B concentrations (125, 300, and 500 nM counterclockwise from top right). **f**, Functionalized surgical mask for the detection of the RBD spike protein (top) and bioluminescence images along the printed pattern in false color (bottom) after exposure to 5 nM (left side of the mask) and 30 nM (right side of the mask) RBD concentration. **g**, Silk-functionalized ink printed on a nitrile glove (top) with the zoom of the pattern (inset), and bioluminescence images along the printed pattern in false color after exposure at various RBD concentrations (50, 12.5,1.25, 0.25 nM from left to right). **h**, Prototype of a small drone carrying a silk sponge for the detection of RBD in contaminated air. **i**, Luminescence response of the 2% RSF 25nM lucKey-lucCageRBD sponge after exposure at vapor with 500pM RBD target against control (water).
