# Supplementary material for: Reshaping de novo protein switches into bioresponsive materials for biomarker, toxin, and viral detection
Source: Adv Mater. Author manuscript; Available in PMC 2026 Nov 2. (PMC13727154; doi:10.1002/adma.202208556)
Supplement: Supplementary file 1 [file NIHMS2209696-supplement-De_Novo_Sensors_FIN_SI.docx]

Supplementary Materials for

Reshaping de novo protein switches into

bioresponsive materials for biomarker, toxin, and viral detection

Luciana d’Amone†, Giusy Matzeu†, Alfredo Quijano-Rubio,

Gregory P. Callahan, Bradley Napier,

David Baker, Fiorenzo G. Omenetto

Correspondence to: dabaker@uw.edu, fiorenzo.omenetto@tufts.edu

**This PDF file includes:**

Materials

Supplementary Text

Figs. S1 to S14

Table S1 to S4

Captions for Data S1 to S14

Materials and Additional Methods

Materials

Sodium carbonate, lithium bromide, and 1X phosphate buffer (PBS) were purchased from Sigma-Aldrich (USA). Ethanol (100%) and Teknova 10% TWEEN-20 solution were purchased from Fisher Scientific. The furimazine substrate was part of the kit Nano-Glow® Luciferase Assay System and was purchased from Promega. Human HER2 / ErbB2 Protein, His Tag (MALS verified) was purchased from Acro Biosystems. LucKey and lucCage constructs (lucCageRBD, lucCageHer2, lucCageHBVα and lucCageBot) were expressed and purified as previously reported ^1^. Monomeric SARS-CoV-2 RBD was expressed and purified as previously described ^2^. The anti-HBV antibody HzKR127-3.2 was purified as previously described ^1^. Botulinum neurotoxin B HcB expressed as previously reported ^3^. All chemicals were used as received and they followed trace metal standard, when possible. Silk cocoons from Bombyx Mori silkworm were purchased from Tajima Shoji Co. (Yokohama, Japan). Deionized water with resistivity of 18.2 MΩ cm was obtained with a Milli-Q reagent-grade water system and used to prepare aqueous solutions. Polystyrene sheets (1.6 mm thickness) used as substrates for printing were purchased from McMaster Carr. 96 well plates (Corning® 96 Well Half-Area Microplate, CLS3693) were purchased from Sigma-Aldrich (USA). Surgical masks (Disposable 3-Ply Masks, 12-888-001A), laboratory nitrile gloves (S79585XS), and PCR tubes were purchased from Fisher Scientific. Simulated saliva (1700-0305) was purchased from Pickering Laboratories.

**Supplementary Methods**

1. Biosensor protein production and purification

The lucCage biosensor proteins lucCageRBD, lucCageHBVα, lucCageHER2, lucCageBot and lucKey were purified as described in Quijano-Rubio et al. 2021. Briefly, glycerol stocks of E. coli Lemo21(DE3) cells (NEB) previously transformed with pET29b+ plasmids encoding the synthesized genes of interest were grown for 24 hours in LB media supplemented with kanamycin. The starter culture was then inoculated at a 1:50 mL ratio in the Studier TBM-5052 autoinduction media supplemented with kanamycin, grown at 37 °C for 2-4 hours, and then grown at 18 °C for an additional 18 h. Cells were harvested by centrifugation at 4000g at 4 °C for 15 min and resuspended in 30 ml lysis buffer (20 mM Tris-HCl pH 8.0, 300 mM NaCl, 30 mM imidazole, 1 mM PMSF, 0.02 mg/mL DNAse). Cell resuspensions were lysed by sonication for 2.5 minutes (5 second cycles). Lysates were clarified by centrifugation at 24,000g at 4 °C for 20 min and passed through 2 ml of Ni-NTA nickel resin (Qiagen, 30250) pre-equilibrated with wash buffer, (20 mM Tris-HCl pH 8.0, 300 mM NaCl, 30 mM imidazole). The resin was washed twice with 10 column volumes (CV) of wash buffer, and then eluted with 3 CV of elution buffer (20 mM Tris-HCl pH 8.0, 300 mM NaCl, 300 mM imidazole). The eluted proteins were concentrated using Ultra-15 Centrifugal Filter Units (Amicon) and further purified by using a SuperdexTM 200 Increase 10/300 GL (GE Healthcare) size exclusion column in Tris Buffered Saline (TBS; 25 mM Tris-HCl pH 8.0, 150 mM NaCl). Fractions containing monomeric protein were pooled, concentrated, snap-frozen in liquid nitrogen, and stored at -80 °C.

2. Bioluminescence imaging

Bioluminescence images were acquired with the Molecular Imaginer® ChemiDoc XRS + Imaging System (Bio-Rad) using the Chemi High Resolution mode. Images were acquired at time intervals of 5 minutes, for a total of 25 minutes.

Imaging of films: For these experiments, RSF films were prepared with the same mix used for printing. Briefly, 20 μL of bio-reactive silk solution at a final concentration of 2 wt% were cast on polystyrene substrates and let dry overnight at room temperature. To analyze the film responsiveness to various target concentrations, films were exposed to target and furimazine. For the experiments with RBD, films were exposed to target in both water and simulated saliva before luminescence readouts.

To check the specificity of the sensing films, interference experiments were carried out using RBD films. These were exposed to the RBD protein (control) or interfering analytes (BoNT/B, anti-HBV antibody, and HER2 ectodomain protein), and the corresponding bioluminescence signal was detected in presence of furimazine. The concentration of the serial dilutions of the analytes was set to 0.32, 1.6, 40 and 200 nM.

Imaging of patterned ink: The same procedure described above was used to test the bioluminescence signals of the silk-based patterns dispensed on laboratory gloves, the jar lid (as viral particle sketches), and on the silicone breast pad.

Imaging of surgical masks: The surgical mask was tested mimicking a real scenario to simulate the use of non-specialized users. In this case, the furimazine substrate solution (2.5 wt%) and target protein solutions (30 nM and 1 nM) were sprayed on the mask, without precise control on the procedure (in terms of sprayed volumes and area of the mask), and the bioluminescence signal was monitored for 25 minutes as previously discussed.

For an easier visualization, all the collected images were processed in false color (reverse Viridis color map reference) and using image processing libraries in Python software. The reverse Viridis color map associates high luminesce readings to yellow hues and low/absent signals to blue hues. The green gradient that developed between the blue and yellow hues accounts for the growth in chemiluminescence readouts. The false color images were post-analyzed using the software Fiji- ImageJ to quantify and relate the intensity variations in luminescence to variations in analyte concentrations.

3. Preparation of silk-based electrospun non-woven mats

The lucKey-lucCageRBD initial stock solutions were diluted in deionized water and mixed for 45 minutes with a plate shaker to enable equilibration. The solution was then combined with a mixture of 7 wt% RSF and 1.2 wt% polyethylene glycol to obtain a final concentration of lucKey-lucCageRBD of 25 nM. A custom-made electrospinning setup was used to fabricate the non-woven mats. Prior to electrospinning, the metal collection circular plate was coated with Teflon spray to facilitate sample removal after electrospinning. 1 mL of solution was then extruded through an 18-gauge needle at a flow rate of 0.90 mL/hr. An applied voltage of 16kV to the needle caused jet initiation and fiber collection on a grounded collector positioned 18.5 cm away. The electrospinning apparatus was contained in an electrically insulated and environmentally controlled enclosure which was maintained at 25% humidity. After electrospinning, the fiber mat was collected, cut in circles (1cm of diameter) with a laser cutter (Trotec Speedy 300) and subsequently exposed to target and furimazine before bioluminescence imaging.

4. Responsiveness of RBD sponges to RBD-spiked vapor

To test the ability of the bio-responsive silk sponges to detect the SARS-CoV-2 RBD protein in air, 25 nM lucKey-lucCageRBD silk sponges were exposed to aqueous vapor with and without 500 pM of RBD target protein. The sponges were placed in a stainless-steel cylindrical chamber kept on a heating plate at 120°C containing a water bath to generate the water vapor and exposed for 20 minutes. To prevent the direct contact of the sponges with the bath, they were placed on a metallic mesh. After the exposure to the vapor, the bioluminescence response of the sponges dissolved in deionized water was acquired for 1 h with a bench top plate reader.

Supplementary Text

Screening of silk-based solutions for HER2 detection

Preliminary studies were carried out to assess the impact of RSF at different concentrations on the lucKey-lucCage protein biosensor functionality and the HER2 protein complex was used as a model. In these experiments, different combinations of lucKey-lucCage at final concentrations of 1 nM and 5 nM were embedded into RSF solutions at 4 wt%, 3 wt%, 2.5 wt%, and 2 wt% (Figure S1). These bio-reactive mixes were tested using the approach described in Methods Section 4.

The lucKey-lucCage biosensing units maintained detecting capabilities after combination with silk fibroin solutions (Figure S1). The chemiluminescent signal intensity was directly proportional to the concentration of the target for all RSF concentration, enabling the detection of HER2 down to nM levels (for all the different combinations, LOD_avg_ 3.5±1.6 nM).

The concentration of RSF influenced the luminescence intensity but did not compromise the overall sensing performances in terms of dynamic range and average LODs. The trend shows that the lower the amount of silk (2 wt%), the higher the luminescence intensities were. Luminescence intensities range from ~1x10^6^ (with silk at 2 wt%) down to ~2.6x10^5^ counts (with silk at 4 wt%), at high target concentrations (125 nM HER2, 5 nM lucKey-lucCage, Figure S1C). The same trend was recorded for solutions embedding 1nM lucKey-lucCage (Figure S1B). The luminescence intensities decreased overall, with a reading of 8x10^4^ counts for silk at 2 wt%, and 1.5x10^4^ counts for silk at 4 wt%. However, the lower amount of lucKey-lucCage did not affect the dynamic sensing range (5-125 nM) and LODs (LOD_avg_ = 3.3±1.4 nM).

Additional testing was carried out on silk-based solutions at 4 wt%, 3 wt%, 2.5 wt%, and 2 wt% embedding 0.5 nM of lucKey-lucCage (Figure S1A). The sensing response for silk solutions at 4 wt% differs from the other mixtures during the detection of low target concentrations (range 0.04-5 nM). The luminescence intensities decreased (down to 3000-8000 counts) even with the highest target concentration (125 nM). The overall detecting performances of 3 wt%, 2.5 wt%, and 2 wt% (0.5 nM lucKey-lucCage) silk-based solutions were preserved, but luminescence counts were lower (8000-38000 counts).

It is likely that silk fibroin interacts with the lucKey-lucCage protein complex through electrostatic and hydrophobic interactions. These types of interactions are involved in the stabilizing effect of silk towards proteins and enzymes in solution, preventing protein-protein interactions and thus aggregation^4^. In this case, higher concentrations of silk fibroin could shield the analyte binding sites of the lucKey-lucCage complex, leading to a drop in bioluminescence intensity.

Bio-reactive sponges and films

After the optimization of the lucKey-lucCage-RSF hybrid in solution, the sensing performances of the complex after incorporation in the solid-state were tested to evaluate the ability of RSF to generate shelf-stable self-standing sensing substrates (Fig. S2-S9). Lyophilized sponges and films were prepared as discussed in the Methods Section 2. Preliminary tests were carried out to verify that the freeze-drying treatment and film casting did not compromise the functionality of the lucKey-lucCage biosensing complex. Free standing RSF formats were prepared using 2 wt% RSF and lucKey-lucCage at concentrations of (1) 5 nM for HER2 and RBD, (2) 25 nM for BoNT/B, (3) 6.25 nM for anti-HBV. Sponges and films were dissolved in water and exposed to various concentrations of analyte to evaluate potential changes in the intensity of chemiluminescent signals, using a microplate reader.

Bioluminescence response showed that the lucKey-lucCage system was stable and responsive both in the sponge (Figure 2b,d,f,h, S2, S3, S4, S5) and film formats (Figure S6-S9), as the chemiluminescent signal intensity increases with target concentrations while achieving detection within 20 minutes. The computed LODs are reported in Table S3.

The chemiluminescent response of 4% and 2% silk sponges (Figure S2-S5) and films (Figure S6A-D, S7F-G, S8B-C, S9B-C) showed a similar trend. For sponges, the amount of RSF directly influenced the luminescence intensity but did not compromise the sensing performance, both in terms of dynamic range and ranges of computed LODs (data reported in Table S4). Luminescence counts were still high for RSF at 4 wt%, reaching values of ~1x10^6^ for HER2, ~3.9x10^5^ for RBD, and ~8x10^5^ for BoNT/B. For RSF film at 4 wt% luminescence counts reached values of ~8.5x10^5^ for HER2, ~3x10^5^ for RBD, for ~4.6x10^5^ BoNT/B, and ~1.9x10^6^ for anti-HBV.

Different concentrations of lucKey and lucCage (1 nM and 5 nM) were tested to analyze their effect on the overall performance of HER2 and RBD detection in sponges and film formats.

The concentration of lucKey-lucCage did not affect the dynamic sensing range (2.5-125 nM) and LOD, i.e., 1.1± 0.1 for lucCageHER2 sponges (Figure S2B, D, F), and 1.2±0.2 for films (Figure S6B, D, F, H). However, it influenced the performances of both the lucCageRBD sponges and films. 1nM of lucKey-lucCageRBD complex in 4 wt% RSF solution (results not shown) did not allow reliable detection due to the high baseline noise. Although the luminescence signal was detectable for 1 nM lucKey-lucCageRBD 2 wt% RSF sponges (Figure S3C-D) and films (Figure S7B-C) the baseline noise was still high.

The best performances were achieved with sponges and films prepared with 5 nM lucKey-lucCageRBD and 2 wt% or 4 wt% RSF solution, which enabled accurate detection of RBD within the sensing range of 20-150 pM. For each analyte, the concentration of lucKey-lucCage was adjusted to optimize the sensing performances of the solid substrates.

The performance of the lucKey-lucCageRBD system towards the Omicron variant of the SARS-CoV-2 Spike RBD target was also tested in a 2% RSF solution. Although the system has not been optimized for the detection of Omicron, the lucKey-lucCageRBD system can detect the variant at nM concentration (Figure S10). Further optimization of the lucKey-lucCage binding energies with the Omicron target could lead to activation of the system at the lower target concentrations.

Bio-reactive films analyzed via chemiluminescence imaging

To speed up the detection method and facilitate sample analysis in non-laboratory settings, the sensing performances of responsive films were evaluated with a bioluminescence camera. Films were casted on white polystyrene substrates using the approach described in Methods Section 2 and the sensing performances of the films were assessed using a chemiluminescent imaging system (Methods Section 6, and Supplementary Methods 2). This detection method was selected since the reading unit can be potentially miniaturized into a portable device to enable delocalized monitoring outside laboratory-controlled conditions ^8-11^.

Imaging of lucKey-lucCageRBD films was achieved with the lucKey-lucCage complex at a concentration of 25 nM (Figure S11) and allowed reaching LODs within the clinical diagnostic range expected for RBD detection (~59 pM). The 25 nM films allowed the monitoring of RBD variations even in simulated saliva down to ~10 pM (Figure 2g), thus supporting the potential use of these sensing platforms as point of care devices. Responsive films were also prepared for HER2 (Fig. 2c), BoNT/B (Fig. 2e), and anti-HBV (Figure 2i) with 25 nM, 50 nM, and 25 nM of lucKey-lucCage, which gave LOD values of ~15 nM, ~8.7 nM, and ~2 nM, respectively.

To assess the stability of the system in harsh conditions, the lucKey-lucCageRBD was electrospun to obtain a non-woven mat that could have applications in air filtration units. The biosensing mix maintained its responsiveness when integrated in an electrospun mat (Fig S11B), and the luminescence signal was still proportional to the concentration of analyte in the pM range.

Bio-responsive tools monitored via chemiluminescence imaging

Bio-responsive patterns were printed or deposited onto different interfaces, including, a silicone breast pad (Fig.3b-c, S12C), jar lid (Fig. 3d-e, S12D), a surgical mask (Fig.3f, S12A), and nitrile gloves (Fig.3g, S12B). Details on the procedure used for printing and imaging are provided in Supplementary Methods 5.

The printing process did not affect the sensing capability of the lucKey-lucCage complex, and the different printed patterns provided a chemiluminescent response proportional to various target concentrations in presence of furimazine substrate (Figure 3, S12). Although the use of different substrates may influence the background noise of the chemiluminescence image, the signal resolution is not compromised.

The analysis of the mask (Figure 3f, S12A) revealed a broad luminescent signal that developed around the whole printed area hampering a precise localization of the chemiluminescent response related to various RBD concentrations, likely due to the lack of control during furimazine and target spraying.

Aiming at the detection of HER2, HER2-RSF hybrid complex were integrated onto a silicone breast pad. As most breast cancers arise from the ductal epithelium, examination of the breast ductal fluid can be used for early detection of breast cancer and to monitor and predict the efficacy of therapies ^12,13^. HER2 is found in nipple fluids at a higher concentration than in blood^14^, and soluble HER-2 (whose normal concentration is in the range of ng/ml) is present in higher concentrations in nipple fluid of patients with tumors^15-17^. Thus, sensing breast pads could represent a breakthrough in the early detection of breast cancer when spontaneous nipple discharge is present, as they could detect whether the discharge is benign or malign. HER2-RSF sensing pads were responsive and able to detect to different concentrations of HER2 within minutes (~20 min) in the nM range (Figure 3c, S12C).

In summary, the results presented in Figure 3 support that the versatility of silk fibroin combined with the accuracy and specificity of the lucKey-lucCage complex enables converting common tools (such as masks and breast pads) into sensing platforms. This strategy paves the way towards responsive devices for multi-analyte detection in biofluids (e.g., salivary analytes). Moreover, these sensing devices can be adapted to other real-life scenarios, enabling the detection of air-borne viruses during sanitary crisis (e.g., SARS-CoV-2), or cancer biomarkers (e.g., HER2, marker of breast cancer) during screening or follow-up examinations. The lucKey-lucCage sensing complex can be further tuned to expand detecting approaches and enable luminescent and fluorescence readouts that may facilitate the translation into readily available miniaturized point of care devices.

Sensing performances of RBD sponges and films during accelerated aging tests

The shelf stability of film and sponge formats was monitored via accelerated aging tests. The RBD detecting formats were prepared and characterized as described in the Methods Section 6. Different compositions in terms of RSF concentration (2 wt% and 4 wt%) and lucKey-lucCage amount (1 nM - only for 2 wt% - and 5 nM) were analyzed over time.

The luminescence intensity recorded for sponges prepared with RSF at 4 wt%, and lucKey-lucCage at 5 nM decreased over time when using 50 nM RBD target concentration, with a variation of ~25% between the first (7 hours) and final reading (4 months, Figure S13A). The overall sensing trend, however, and the LODs were preserved over time (Figure S13C). The same trends were recorded for RSF-based sponges (2 wt%) embedding 1nM or 5 nM of lucKey-lucCage. Both sensitivity and dynamic ranges were fully preserved over time, and the LODs values (LOD_avg_= 22±4.3 pM) were stable for the different types of sponges over 4 months (Figure S13E).

Films at 2 wt% silk, 5 nM lucKey-lucCage analyzed at 150 nM of RBD concentration were found to be more stable, as luminescence intensities varied of ~4% between the first and the last reading (Figure S13B). The overall sensing trend and the LODs were preserved over time (Figure S13D). Similar responses were recorded for RSF-based films (4 wt%, 2 wt%) embedding 5 nM or 1 nM of lucKey-lucCage. The sensing performances (both sensitivity and dynamic ranges) were fully preserved over time, the LODs were stable at an average value of 14±4 pM (Figure S13F).

Some of the computed LOD values listed in Figure S13E-F are outliers (Figure S13E silk 2% lucKey-lucCage 5 nM, 44 pM; Figure S10F, silk 2% lucKey-lucCage 5 nM, 50 pM; silk 4% lucKey-lucCage 5 nM, 44 pM). These unusual higher LODs can be dictated by variations in film dissolution before analysis. Nevertheless, the overall stability of the solid protein complex at 60°C for 4 months suggests that the system is robust and preserves the detecting performances in non-refrigerated conditions, which is an essential feature for point of care platforms, yet difficult to achieve with protein-based devices.

The stability of HER2-RSF hybrid sponges was re-tested after storage at room temperature (20-25ºC) over 1 year. Despite the lucKey-lucCage sensing components are typically refrigerated at -80ºC, the integration in the RSF matrix preserved their sensing capabilities (Fig. S14). The HER2-RSF sponges after dissolution and exposure to target and furimazine lead to luminescence signals proportional to the concentration of analytes and enabled detection within the nM range (LOD = 5nM).

Fig. S1. Effect of silk fibroin concentration on HER2 detection.

Chemiluminescence intensities at various target and regenerated silk fibroin concentrations at 0.5 nM (A), 1 nM (B), and 5 nM (C) of lucKey-lucCageHER2.

Fig. S2. Luminescence characterization of HER2-responsive silk sponges.
Luminescence kinetics (A, C, E) and corresponding normalized luminescence (B, D, F) as a function of HER2 target concentration of 4% silk fibroin sponges embedding 1 nM (A, B) or 5 nM (C, D) of lucKey-lucCageHER2, and 2% silk fibroin embedding 1 nM (E, F) of lucKey-lucCageHER2 after exposure at various concentrations of HER2 ectodomain protein (0.04-125 nM). Detecting performances were extrapolated from the luminescence kinetics at 102 minutes (B, D) or 51 minutes (F).

Fig. S3. Luminescence characterization of RBD-responsive silk sponges.
Luminescence kinetics (A, C) and corresponding normalized luminescence (B, D) as a function of SARS-CoV-2 RBD target concentration of 4% silk fibroin sponges embedding 5 nM (A, B) of lucKey-lucCageRBD, and 2% silk fibroin embedding 1 nM (C, D) of lucKey-lucCageRBD after exposure at various concentration of RBD (0.4-150 pM). Detecting performances were extrapolated from the luminescence kinetics at 51 minutes (B, D).

Fig. S4. Luminescence characterization of BoNT/B-responsive silk sponges.
Luminescence kinetics (A) and corresponding normalized luminescence as a function of BoNT/B target concentration (B) of 4% silk fibroin sponges embedding 25 nM of lucKey-lucCageBot after exposure at various concentrations of BoNT/B (0.064-200 nM). Detecting performances were extrapolated from the luminescence kinetics at 51 minutes (B).

Fig. S5. Luminescence characterization of anti-HBV antibody-responsive silk sponges.	Normalized luminescence as a function of anti‑HBV antibody concentration of 2% silk fibroin sponges embedding 6.25 nM of lucKey-lucCageHBVα after exposure at various concentrations of anti-hepatitis B virus antibody (0.064-40 nM). Detecting performances were extrapolated from the luminescence kinetics at 51 minutes.

Fig. S6. Luminescence characterization of HER2-responsive silk films.
Luminescence kinetics (A, C, E, G) and corresponding normalized luminescence as a function of HER2 concentration (B, D, F, H) of 4% silk fibroin films embedding 1 nM (A, B) or 5 nM (C, D) of lucKey-lucCageHER2, and 2% silk fibroin embedding 1 nM (E, F) or 5 nM (G, H) of lucKey-lucCageHER2 after exposure at various concentrations of HER2 ectodomain protein (0.04-125 nM). Detecting performances were extrapolated from the luminescence kinetics at 102 minutes (B, D, F) or 51 minutes (H).

Fig. S7. Luminescence characterization of RBD-responsive silk films.
(A) Normalized luminescence as a function of RBD concentration of lucKey-lucCageRBD in 2% silk solution after exposure at various concentrations of RBD (2-1250 pM) (in liquid control). Luminescence kinetics (B, D) and corresponding normalized luminescence as a function of RBD concentration (C, E) of 2% silk fibroin films at 1 nM (B, C) or 5nM (D, E) and 4% silk fibroin films at 5nM (F, G) of lucKey-lucCageRBD after exposure at various concentrations of RBD (0.4-150 pM). Detecting performances were extrapolated from the luminescence kinetics at 120 (A), 20 (C), or 51 minutes (E, G).

Fig. S8. Luminescence characterization of BoNT/B-responsive silk films.
(A)Normalized luminescence as a function of BoNT/B concentration of lucKey-lucCageBot in 4% silk solution after exposure at various concentrations of BoNT/B (0.032-200 nM) (in liquid control) . Luminescence kinetics (B, D) and corresponding normalized luminescence (C, E) as a function of BoNT/B concentration of 4% silk fibroin films (B, C) and 2% silk fibroin (D, E) embedding 25 nM of lucKey-lucCageBot after exposure at various concentrations of BoNT/B (0.064-200 nM). Detecting performances were extrapolated from the luminescence kinetics at 120 (A) and 24 minutes (C, E).

Fig. S9. Luminescence characterization of anti-HBV antibody-responsive silk films.
(A) Normalized luminescence as a function of anti-hepatitis B virus antibody concentration of lucKey-lucCageHBV in 2% silk solution after exposure at various concentrations of anti-hepatitis B virus antibody (1.6-200 nM) (in liquid control). Luminescence kinetics (B, D) and corresponding normalized luminescence (C, E) as a function of target concentration of 4% (B, C) and 2% (D, E) silk fibroin films embedding 6.25 nM of lucKey-lucCageHBV after exposure at various concentrations of anti-hepatitis B virus antibody (0.064-200 nM for A-B, 0.064-40 nM for D-E). Detecting performances were extrapolated from the luminescence kinetics at 120 (A) and 24 minutes (C, E).

**Fig. S10. Responsiveness of RBD-sensing complex to the Omicron variant target.** Luminescence kinetics (A) and corresponding normalized luminescence (B) as a function of SARS-CoV-2 (Omicron variant) concentration of a 2% silk solution with 10 nM lucKey-lucCageRBD. Detecting performances were extrapolated from the luminescence kinetics at 120 minutes (B).

Fig. S11.Bioimaging characterization of RBD-responsive silk films and non-woven mat.
(A) Chemiluminescent signals plotted as the average pixel intensities of the imaged sensing areas and respective chemiluminescent responses in false color (insert) of 25 nM lucKey-lucCageRBD films after exposure at furimazine and various target concentrations in water. (B) Photograph of an electrospun mat (top left), corresponding SEM micrograph (top right), and bioluminescence response (bottom) in false color in presence of increasing concentrations of RBD (2-1250 pM from left to right).

**Fig. S12. Bioimaging of functional biomaterials**

(A) Bioluminescence images of the surgical mask along the printed pattern in false color after exposure to 5 nM (left side of the mask) and 30 nM (right side of the mask) RBD concentration (top) and luminescence response reported as gray value (bottom). (B) Bioluminescence images of a nitrile glove along the printed pattern in false color (top) and luminescence response at various RBD concentrations (50, 12.5,1.25, 0.25 nM from left to right) reported as gray value (bottom). (C) Bioluminescence images of the functionalized breast pad in false color (right) and luminescence response at various Her2 target concentrations (50 nM, 250 nM, 500 nM and 12.5 nM clockwise from the top left) reported as gray value (left). Higher target concentration and merging of multiple drops during sample analysis lead to increasing luminescence intensity. (D) Bioluminescence images of the functionalized jar lid in false color (top) and luminescence response at different BoNT/B concentrations (500, 300,125 nM from left to right) reported as gray value (bottom).

Fig. S13. Accelerated aging tests of RBD-responsive silk sponges and films.
Luminescence kinetics (A, B) and corresponding normalized luminescence (C, D) of 4% silk fibroin (A, C) and 2 % silk fibroin sponges (B, D) embedding 5 nM of lucKey-lucCageRBD after exposure to 50 pM (A) or 150 pM (B) of RBD at different incubation times. Tables report the corresponding LODs for sponges (E) and films (F).

**Fig. S14. Stability studies of HER2-responsive silk sponges**

Luminescence kinetics (A) and corresponding normalized luminescence (B) of 2% silk fibroin sponges embedding 5 nM of lucKey-lucCageHER2 stored at room temperature for one year after exposure at furimazine and various concentrations of HER2 (0.2-125nM).

Table S1. Initial concentration of stock solutions.

Table S2. Composition of sponges and films.

Table S3. LODs of silk sponges and films.

Table S4. Sensing ranges and LODs for sponges with various silk concentration.

**References**

1	Quijano-Rubio, A. *et al.* De novo design of modular and tunable protein biosensors. *Nature* **591**, 482-487 (2021).

2	Walls, A. C. *et al.* Structure, Function, and Antigenicity of the SARS-CoV-2 Spike Glycoprotein. *Cell* **183**, 1735, doi:10.1016/j.cell.2020.11.032 (2020).

3	Jin, R., Rummel, A., Binz, T. & Brunger, A. T. Botulinum neurotoxin B recognizes its protein receptor with high affinity and specificity. *Nature* **444**, 1092-1095, doi:doi:10.1038/nature05387 (2006).

4	d'Amone, L., Matzeu, G. & Omenetto, F. G. Stabilization of Salivary Biomarkers. *ACS Biomater Sci Eng* **7**, 5451-5473, doi:10.1021/acsbiomaterials.1c01138 (2021).

5	Arts, R. *et al.* Detection of antibodies in blood plasma using bioluminescent sensor proteins and a smartphone. *Analytical chemistry* **88**, 4525-4532 (2016).

6	England, C. G., Ehlerding, E. B. & Cai, W. NanoLuc: a small luciferase is brightening up the field of bioluminescence. *Bioconjugate chemistry* **27**, 1175-1187 (2016).

7	Xue, L., Yu, Q., Griss, R., Schena, A. & Johnsson, K. Bioluminescent Antibodies for Point‐of‐Care Diagnostics. *Angewandte Chemie International Edition* **56**, 7112-7116 (2017).

8	Calabretta, M. M., Álvarez-Diduk, R., Michelini, E., Roda, A. & Merkoçi, A. Nano-lantern on paper for smartphone-based ATP detection. *Biosensors and Bioelectronics* **150**, 111902 (2020).

9	Griss, R. *et al.* Bioluminescent sensor proteins for point-of-care therapeutic drug monitoring. *Nature chemical biology* **10**, 598-603 (2014).

10	Cevenini, L. *et al.* Exploiting NanoLuc luciferase for smartphone-based bioluminescence cell biosensor for (anti)-inflammatory activity and toxicity. *Analytical and bioanalytical chemistry* **408**, 8859-8868 (2016).

11	Kim, H. *et al.* Smartphone-based low light detection for bioluminescence application. *Scientific reports* **7**, 1-11 (2017).

12	Evron, E. *et al.* Detection of breast cancer cells in ductal lavage fluid by methylation-specific PCR. *Lancet* **357**, 1335-1336, doi:10.1016/s0140-6736(00)04501-3 (2001).

13	Petrakis, N. L. Nipple aspirate fluid in epidemiologic studies of breast disease. *Epidemiol Rev* **15**, 188-195, doi:10.1093/oxfordjournals.epirev.a036104 (1993).

14	Brooks, M. in *Cancer Epidemiology*	pp 307-321 (SpringerLink, 2021).

15	Kuerer, H. M. *et al.* High and differential expression of HER-2/neu extracellular domain in bilateral ductal fluids from women with unilateral invasive breast cancer. *Clin Cancer Res* **9**, 601-605 (2003).

16	Lang, D. J. E. & Kuerer, H. M. Breast Ductal Secretions: Clinical Features, Potential Uses, and Possible Applications:. [*http://dx.doi.org/10.1177/107327480701400405*](http://dx.doi.org/10.1177/107327480701400405), doi:10.1177_107327480701400405 (2007).

17	Inaji, H. *et al.* ErbB-2 protein levels in nipple discharge: role in diagnosis of early breast cancer. *Tumour Biol* **14**, 271-278, doi:10.1159/000217839 (1993).
